# “One-stop” combined percutaneous left atrial appendage and atrial septal defect closure in atrial fibrillation: safety and feasibility from a single-center cohort

**DOI:** 10.3389/fcvm.2025.1579786

**Published:** 2025-06-19

**Authors:** Gaofeng Wang, Qiqiang Jie, Kailing Xu, Jie Luo, Jing Dong, Dujiang Xie, Ling Zhou

**Affiliations:** ^1^ Department of Cardiology, Nanjing First Hospital, Nanjing Medical University, Nanjing, Jiangsu, China; ^2^ Department of Urology, Huai'an Second People’s Hospital Affiliated to Xuzhou Medical University, Huai'an, Jiangsu, China; ^3^ Department of Cardiovascular Ultrasound, Nanjing First Hospital, Nanjing Medical University, Nanjing, Jiangsu, China

**Keywords:** atrial fibrillation, atrial septal defect, congenital heart disease, left atrial appendage closure, device

## Abstract

**Background:**

Patients with atrial fibrillation (AF) and atrial septal defect (ASD) face elevated thromboembolic risks, yet evidence on combined left atrial appendage closure (LAAC) and ASD closure remains limited. We aimed to assess the feasibility and safety of a “one-stop” strategy for simultaneous LAAC and ASD closure.

**Methods:**

A retrospective analysis included 40 patients with non-valvular AF and ASD undergoing combined procedures (2016–2024). Procedural success, complications, and long-term outcomes (mean follow-up: 1,194.3 days) were analyzed.

**Results:**

All procedures were technically successful. No major complications (stroke, device embolization, or death) occurred during follow-up. Peri-device leak (PDL) was observed in 19 patients (47.5%), with only one case of device-related thrombus (resolved with anticoagulation).

**Conclusion:**

The “one-stop” approach is a safe and effective strategy for stroke prevention in AF patients with ASD, particularly those unsuitable for long-term anticoagulation.

## Introduction

Atrial Septal Defect (ASD) is the most common congenital heart disease in adults, accounting for 25%–30% of new diagnoses (1). ASD induced chronic volume overload leads to right atrial enlargement and electrical remodeling, creating a substrate for AF initiation and perpetuation. Concurrently, AF exacerbates atrial dysfunction, further increasing thromboembolic risks. According to the 2024 ESC Guidelines for the Management of Atrial Fibrillation (AF) (2), patients with ASD are at a significantly higher risk of developing AF due to atrial remodeling and increased atrial pressure. Previous studies showed that AF is more common in ASD patients, especially those elderly people with large defects. The occurrence rates of AF in patients with ASD aged >40 and >60 years are approximately 21 and 52%, respectively (3, 4). Furthermore, the guidelines emphasize that AF in ASD patients is associated with a higher risk of thromboembolic events, particularly stroke, underscoring the importance of early intervention. Compared to surgery, device closure is a better treatment for most patients with a secundum of ASD with lower complication rates and shorter hospital stay and recovery (5). Since over 90% of thrombi in patients with non-valvular atrial fibrillation originate from the left atrial appendage (LAA), transcather closure of LAA has become an effective stroke prevention strategy, which is used as an alternative to long-term oral anti-coagulation therapy (6). While transcatheter ASD closure and LAAC are established individually, simultaneous procedures remain understudied. Potential advantages, such as avoiding repeated transseptal punctures and reducing healthcare costs warrant systematic evaluation. This study addresses this gap by evaluating the feasibility and safety of a one-stop approach.

## Methods

### Patients selection

We retrospectively enrolled patients who underwent ASD occlusion and LAAC at a single tertiary Center (Nanjing First Hospital, Nanjing, China) between January 2016 and June 2024. A patient meets the following criteria to be eligible for the combined procedure: (1). Transthoracic echocardiography (TTE) shows clear evidence for ASD occlusion. (2). Non-valvular AF. (3). CHA2DS2-VASc score ≥2, and/or HAS-BLED score ≥3 or being contraindicated to long-term oral anticoagulants (OACs) or the patient refuses to take long-term OACs despite extensive explanations. Exclusion criteria were as follows: (1). ASD requiring surgical repair or combined with other diseases requiring thoracotomy surgery. (2). LA (Left atrial) or LAA thrombus. (3). Severe heart failure (New York Heart Association class IV). (4). Severe renal or hepatic insufficiency. (5). The patients refused to accept the combined occlusion. Written consent was obtained from all the participants before the procedures. This single-center retrospective cohort study was approved by the Institutional Review Board of Nanjing First Hospital.

### One-stop procedure

**Figure 1:**
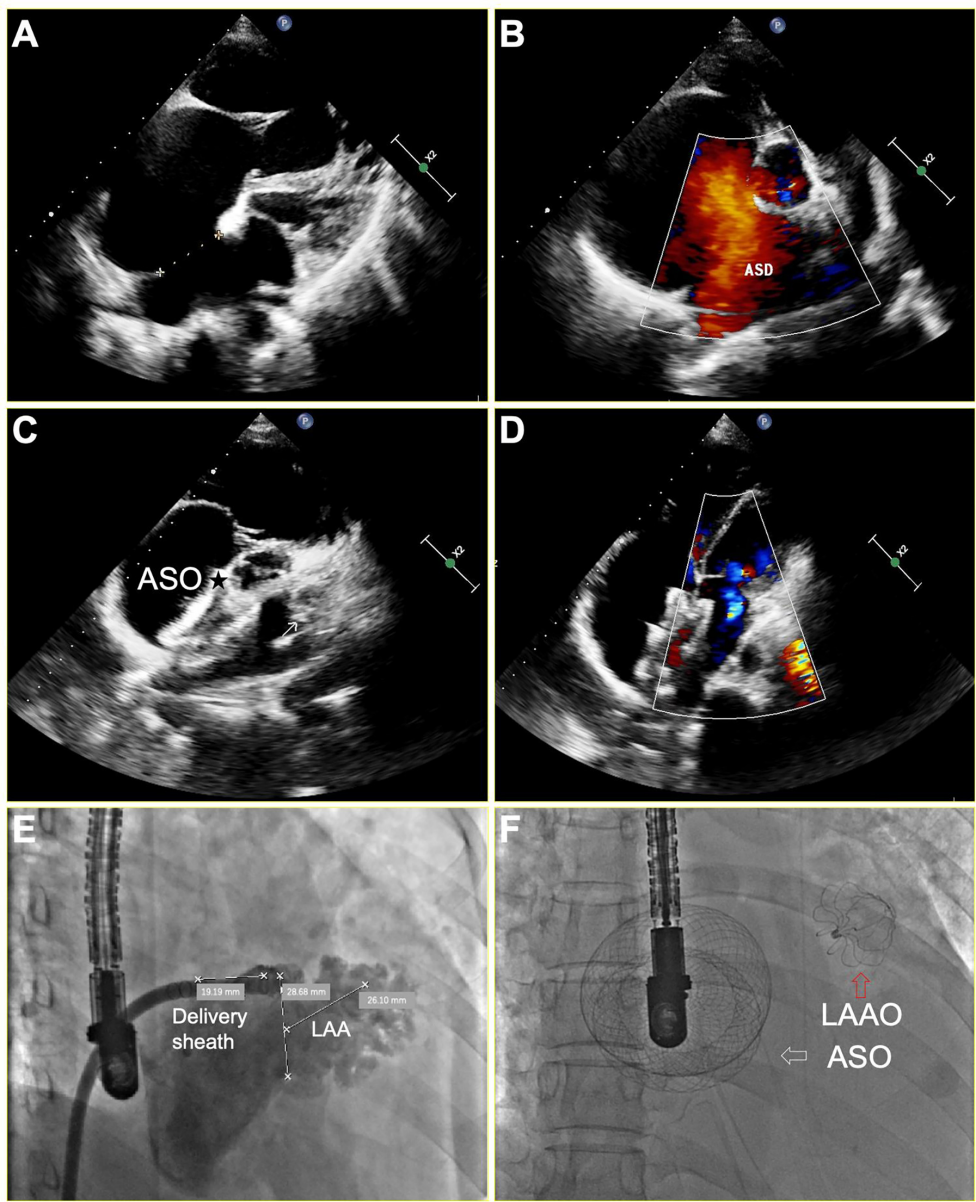
TEE and x-ray imaging of successful LAA and ASD occlusion for a patient with persistent AF and a larger ASD. **(A,B)** ASD measuring 33 mm in diameter, resulting in a significant left-to-right shunt in the color-Doppler analysis; **(C,D)** Disappearance of complete shunt after a 46 mm ASD occlusion device (asterisk) implantation; **(E)** A fluoroscopic illustration of the LAA positions and shapes before operation. **(F)** A fluoroscopic illustration of successful LAAC and ASD occlusion, white arrow indicates the ASO, while red arrow indicates the LAAO. ASD, atrial septal defect; ASO, atrial septal occluder; LAAO, left atrial appendage occluder.

The right femoral vein was punctured under local anesthesia, then right and left heart catheterization was performed to measure the pressure of pulmonary artery, right ventricle, right atrium and left atrium. Pulmonary vascular resistance (PVR) and pulmonary to systemic blood flow (Qp/Qs) ratio were calculated by the Fick equation (7). Once the patient met the indications for closing ASD as previously described (5), general anesthesia was given to perform the following one-stop procedure. The transesophageal echocardiogram (TEE) was performed to assess the presence of thrombi in LAA and measure the size and length of LAA at 0°, 45°, 90°, and 135°. The LAA was closed via conventional method using a WATCHMAN (Boston Scientiﬁc, MA, USA) or LAmbre (Xianjian Technology Co., Shenzhen, China) device. The details regarding LAAC and characteristics of devices were as previously described (8, 9). After successful closure of the LAA, subsequent occlusion of the ASD was performed as previously described (1). Two devices were used: the SHSMA ASD occluder (Shape Memory Alloy Co., shanghai, China), and the Amplatzer ASD occluder (St. Jude Medical, Golden Valley, MN). Figure 1 depicts concurrent LAAC with the WATCHMAN™ device and ASD occlusion under TEE guidance in a patient with persistent AF and ASD.

### Postoperative antithrombotic treatment

All patients were given the OACs (warfarin, dabigatran, or rivaroxaban) for 3 months after discharge. Those with relative contraindications to OAC received a 3-month regimen with frequent monitoring.Then a TEE or cardiac computed tomography angiography (CCTA) was performed to assess the presence of residual peri-device flow and the formation of device-related thrombus (DRT). If the LAA was successfully occluded, as defined by a residual flow of less than 5 mm around the device, the OACs would be stopped and replaced by double antiplatelet therapy (DAPT) until 6 months after the one-stop procedure. Thereafter, aspirin was taken another 6 months and stopped if there were no concomitant diseases requiring long-term use of aspirin.

### Follow-up visit

All participants were followed up at an interval of the first, third, and sixth months. TEE and CCTA were performed to assess the position of devices, remnant shunts and DRT. Long-term follow-up for device-related complications and the presence of stroke, transient ischemic attack (TIA) or other thromboembolism was carried out.

### Statistical analysis

Statistical analyses were carried out using Stata Statistical Software for Professionals (version 18; Stata Corp, College Station, TX). Kolmogorov–Smirnov test was used to assess for the normal distribution of the data. Variables with a normal distribution are described as mean ± standard deviation (SD). Skewed distributed data are expressed as median (interquartile range). Categorical variables are described with counts and percentages. Given the descriptive nature of this article, no between-groups comparisons were performed.

## Results

### Baseline clinical characteristics

Forty-seven patients with ASD underwent LAAC in our center during this period. Three patients without AF who received LAAC for the purpose of “primary primary” prevention were excluded. Four patients who underwent LAA and ASD closure in sequential stage were also excluded. A total of 40 remaining patients were included in this study. The baseline clinical characteristics are summarized in Table 1. Among these patients, 27 (67.5%) were females, and the mean age was 66.4 years (range, 42–85 years). The mean CHA2DS2-VASc score was 3.1 and 5 patients (12.5%) had previous stroke, TIA or systemic embolism. The mean HAS-BLED score was 1.8. There were 7 patients suffered from paroxysmal episodes of AF, and 33 patients had persistent episodes.

**Table 1:** Demographic and clinical characteristics of participants.

Variables	*n* = 40
Demographics	
Age (years)	66.4 ± 9.5
Female [*n* (%)]	27 (67.5)
BMI (kg/m^2)^	25.3 ± 3.2
SBP (mmHg)	127.5 ± 18.0
DBP (mmHg)	80.4 ± 13.9
Medical history	
Paroxysmal AF [*n* (%)]	7 (17.5)
Congestiev HF [*n* (%)]	21 (52.5)
Hypertension [*n* (%)]	18 (45.0)
DM [*n* (%)]	4 (10.0)
Ischemic stroke/TIA [*n* (%)]	5 (12.5)
CAD [*n* (%)]	8 (20.0)
CHA_2_DS_2_-VASc score	3.1 ± 1.6
HAS-BLED score	1.8 ± 0.8
Clinical chemistry	
NT-proBNP (pg/ml)	1,610.7 (828.6, 3,051.1)
Creatinine (µmol/L)	75.5 ± 19.2
Haemoglobin (g/dl)	135.1 ± 19.2
Platelet count (×10^9^/L)	159.7 ± 55.7
Pre-procedural TTE	
LV ejection fraction (%)	59.7 ± 5.2
ASD diameter (mm)	20.3 ± 9.2
LA dimension (mm)	50.3 ± 5.5
Moderate-to-severe MR [*n* (%)]	10 (25.0)
Moderate-to-severe TR [*n* (%)]	27 (67.5)
PASP (mmHg)	56.2 ± 12.8

Values are represented using the mean ± SD and *n* (%).

ASD, atrial septal defect; AF, atrial ﬁbrillation; BMI, body mass index; LA, left atrium; LAA, left atrial appendage; LVEF, left ventricular ejection fraction; OAC, oral anticoagulant; TIA, transient ischaemic attack.

### Procedure details

Procedural details were presented in Table 2. All 40 patients successfully completed the combined procedure. In addition to the ASD and LAA closure, radiofrequency ablation was also performed in 6 patients at the same time. Of the LAAC procedures, 29 (72.5%) patients were implanted with WATCHMAN and 11 (27.5%) patients with LAmbre, respectively. ASD occlusion was performed after LAAC. The mean ASD diameter was 20.3 ± 9.2 mm (range 6–38 mm, median 21 mm). 29 (72.5%) patients were implanted with SHSMA and 11 (27.5%) patients with Amplazer occluder, respectively. The mean size of device was 28.6 ± 9.9 mm (range 10–46 mm, median 28 mm). No major perioperative complications (e.g., tamponade, thrombosis, death) occurred during hospitalization (mean stay: 4.7 ± 1.8 days). Nine patients (22.5%) had a small residual shunt (<5 mm) after LAAC, none large residual shunt (≥5 mm) occurred.

**Table 2:** The periprocedural parameters of the “one-stop” procedure.

Procedural parameters	*n* = 40
Transcatheter ASD closure	
SHSMA occluder [*n* (%)]	29 (72.5)
Amplazer [*n* (%)]	11 (27.5)
Size of device (mm)	28.6 ± 9.9
Successful deployment	40 (100)
Acute procedural complication	0
Transcatheter LAA closure	
TEE	
LAA width(mm)	23.5 ± 2.5
LAA depth(mm)	27.5 ± 2.6
LAA ostium diameter	24.7 ± 2.7
LAA working depth	26.8 ± 2.6
Implanted devices	
LAmbre	11 (27.5)
WATCHMAN	29 (72.5)
Implantation success (%)	100
Procedural complications [*n* (%)]	
Pericardial effusion requiring intervention	0
Major vascular complication	0
Procedure-related stroke	0
Device embolization	0
In-hospital death	0
Peri-device leak, *n* (%)	
>5 mm	0
3–5 mm	1 (2.5)
<3 mm	8 (20.0)

### Follow-up visit

The long-term visit results are presented in Table 3. No patient lost contact during a follow-up of 1,194.3 ± 671.5 days. No patients experienced strokes, TIA, or other thromboembolisms. Moreover, the procedure did not cause any other severe complications, such as tamponade, stroke, or pulmonary vein stenosis. Follow-up TEE and CCTA data at 3 months were available in all the patients. In 21 cases (52.5%) there was no peri-device leak (PDL) detectable by CCTA or TEE. A leak with a width of less than 3 mm was present in 17 cases (42.5%), and a leak width of 3–4.9 mm was present in two cases (5.0%). Among patients receiving the Watchman device, 15 cases (51.7%) demonstrated PDL, with 14 patients exhibiting <3 mm leaks and 1 patient showing 3–5 mm leaks. In contrast, the LAmbre occluder group had 4 documented PDL cases (36.4%), comprising 3 patients with <3 mm leaks and 1 patient with 3–5 mm leaks. None were found to have a severe leak more than 5 mm in width. One patient was diagnosed with an LAA device thrombus during follow-up TEE which dissolved after maintain OAC for another three months. No late device embolization, displacement of devices or pericardial effusions occurred.

**Table 3:** Follow-up outcomes.

Outcomes	*n* = 40
Days from device implantation	1,194.3 ± 671.5
Ischaemic stroke	0
Major haemorrhage	0
Intracranial haemorrhage	0
Digestive tract haemorrhage	0
Epistaxis	0
Unexplained anaemia requiring transfusion	0
Death	0
Imaging modality (%)	
TEE	40
CT	40
Device migration, *n* (%)	0
Device-related thrombus, *n* (%)	1 (2.5)
Late device embolization, *n* (%)	0
Peri-device leak (any), *n* (%)	
>5 mm	0
3–5 mm	2 (5.0)
<3 mm	17 (42.5)
PDL in WATCHMAN, *n* (%)	
<3 mm	14 (48.3)
3–5 mm	1 (3.4)
PDL in LAmbre, *n* (%)	
<3 mm	3 (27.3)
3–5 mm	1 (9.1)

## Disscusion

### Main findings of this study

To the best of our knowledge, this is the largest sample size study conducted to investigate the feasibility and safety of performing ASD and LAA closure in the same setting. Our study shows that LAA and ASD closure can be safely performed in the same setting. This “one-stop” procedure might be an ideal treatment strategy to prevent stroke and other thromboembolism for patients with both NVAF and ASD.

### Possible reasons and comparison with previous studies

Because of increased atrial size, anatomical and electrical remodeling of the atrium, patients with ASD are more likely to develop AF. A Swedish long-term follow-up study (the mean follow-up was 22 years) has showed that the risk of developing AF was 22.26 times higher (95% confidence interval, 14.72–33.68) in patients with ASD than matched control subjects (10). Moreover, the risks for ischemic stroke, heart failure, and death were significantly higher in patients with ASD than control subjects. Hence, it is necessary to close ASD in patients with AF as early as possible.

The optimal management of AF in patients with ASD remains a topic of debate. According to the 2024 ESC Guidelines, rhythm control strategies, such as radiofrequency ablation, may be less effective in ASD patients due to the high recurrence rate of AF, particularly in those with significant atrial enlargement (2). Instead, the guidelines recommend a comprehensive approach that includes stroke prevention strategies, such as LAAC, especially in patients with contraindications to long-term anticoagulation. This aligns with our study's findings, demonstrating the feasibility and safety of combining LAAC with ASD closure in a single procedure. Prior to the transcatheter closure of ASD, it is worth considering whether to close LAA in the same setting. There are several advantages of closing LAA and ASD simultaneously. Firstly, transseptal puncture is usually not necessary in patients with existing ASD. On the contrary, it is a great challenge for transseptal puncture in those previously treated with a large ASD device. Secondly, transcatheter ASD closure employ similar equipment, delivery sheath and vascular approach to LAAC. Performing both closures in the same setting obviously reduces the hospitalization costs. Thirdly, the procedure time and hospital stay are greatly shortened if ASD closure and LAAC are carried out together which improves medical experience. Thus, it is reasonable to perform LAAC and ASD occlusion at the same time.

So far, there are few studies indicating the feasibility and safety of combination of LAAC and transcatheter ASD closure. In 2014, the first report on the safety and feasibility of LAAC and transcatheter closure of ASD was published by Gafoor et al (11). They performed transcatheter ASD closure and LAAC in the same setting in three patients, and one patient had access-site hematoma. However, the sample size was too small and follow-up data was lacking. In a study by Yu et al. (12), the long-term safety and efficacy of combined percutaneous LAA and PFO/ASD closure was firstly demonstrated. Over a 6-year period, the authors compared the long-tern outcomes of 330 patients who underwent only LAAC with 30 patients (PFO/ASD: 25/5) who underwent the combined LAA and PFO/ASD closure at a single center. For the patients who underwent the combined LAA and PFO/ASD closure, only 1 (3.3%) device thrombi and 1 bleeding events were found. This study also demonstrated that the observed annual rate of thromboembolic and bleeding events was significantly decrease when compared to the expected thromboembolic and bleeding events in this set of patients during 823.0 ± 543.7 days follow up. However, in this study, only 5 ASD patients were included and ASD closure did not perform at the same time with LAAC but was scheduled at 48.9 ± 3.5 days after LAAC.They reported similar safety in staged LAAC/ASD closure, but their 3.3% DRT rate exceeds ours (2.5%), and their mean follow-up was shorter (2.3 vs. 3.3 years). Accordingly, the safety and feasibility could not be extrapolated to a combined percutaneous closure of LAA and ASD simultaneously. This treatment strategy may also decrease patient's willingness for occlusion and increase economic burden. A study by Leong et al. (13) reported combined LAA and ASD closure in elderly patients with a significant ASD and AF. The aim of this study was to investigate the effects of LAA and ASD closure on six elderly patients with AF and ASD. Four patients received a simultaneous operation, whereas two individuals underwent a staged process. All patients had successful procedures, and no device-related thrombosis or erosion was recorded. However, the sample size of this study was small (6 patients) and both procedures were done in sequential stage in two patients. The study conducted by Zhang et al (14). Evaluated the safety and effectiveness of combining ASD occlusion and LAA closure simultaneously. 49 patients, including 24 ASD and 25 PFO, were enrolled to perform the combined procedure, and successfully completed the combined occlusion. In two patients, TEE demonstrated occluder thrombosis at 45–60 days follow up, but the thrombus was resolved at 6 months when the anticoagulant treatment scheme was adjusted. There were no strokes, TIAs, or other thromboembolisms during the follow-up period. Compared with the results reported by Zhang et al., the patients enrolled in our study showed larger ASD (20.1 VS 14.5 mm) and LA dimension (51.5 vs. 44.5), and the total postoperative serious adverse events (SAE) was similar. Our 100% procedural success rate aligns with Zhang et al, yet our cohort included larger ASDs (20.3 mm vs. 14.5 mm), suggesting broader applicability. The absence of stroke events over 3-year follow-up surpasses the 2.1% annual risk predicted by CHA2DS2-VASc scores, underscoring the strategy's efficacy. The PROTECT-AF trial reported 3.0% adverse event (stroke, systemic embolism and cardiovascular death) rates in LAAC patients (15), whereas our cohort had zero events, suggesting combined ASD closure may mitigate thromboembolic risks through hemodynamic improvement.

Our study shows a 100% procedural success rate with no deaths and no major complications. One patient was diagnosed with DRT during follow-up TEE which dissolved after maintain OAC for another three months. The higher PDL incidence observed in our cohort may be explained by the implementation of CT surveillance protocols, given CT's established superiority over TEE in detecting subtle PDL. Supporting this hypothesis, Korsholm et al (16) conducted a rigorous cross-modality comparative study where simultaneous cardiac CT and TEE assessments at 3-month post-LAAC revealed a nearly twofold higher PDL detection rate with CT (61% vs. 32%, respectively). In our study, patients receiving the Watchman device demonstrated a significantly elevated PDL incidence relative to the LAmbre occluder group (51.7% vs. 36.4%). This differential performance may be attributed to LAmbre's lobe-disc configuration, which enhances anatomical sealing through dual-layer endothelialization *—* the distal lobe anchors within trabeculated regions while the proximal disc bridges irregular ostial contours. Mechanistically, this dual-occlusive design achieved superior anatomical closure rates (Amulet IDE Trial: 37.0% vs. Watchman 53.9% at 45 days (17) by simultaneously addressing endocardial apposition and ostial coverage, whereas single-occlusive devices primarily rely on endothelial overgrowth over a porous scaffold. Although PDL were observed in 47.5% of cases, none exceeded 5 mm, and no reinterventions were performed. The absence of thromboembolic events underscores the potential safety of conservative management for minor leaks in this population. This aligns with studies suggesting that minor PDLs (<5 mm) do not significantly increase stroke risk (16, 18). To reduce the PDL incidence, we propose optimizing device sizing using preprocedural 3D imaging and prioritizing lobe-and-disc devices for high-risk anatomies. The integrated “one-stop” strategy demonstrates significant cost-effectiveness, achieving an approximately 30% reduction in hospitalization expenses according to institutional billing analyses. This economic advantage is primarily driven by the elimination of redundant procedural expenditures, including repeated anesthesia administration, transesophageal echocardiography (TEE), vascular access procedures, and laboratory testing. Furthermore, the consolidated protocol substantially mitigates patient discomfort while reducing cumulative procedural risks inherent to multiple interventions. Future studies should explore combined ASD closure and primary prevention LAAC in high-risk populations, particularly given the technical challenges of subsequent transseptal access post-ASD closure. Such a strategy may preemptively mitigate thromboembolic risks in patients predisposed to AF development.

### Limitations of the study

Several methodological limitations warrant consideration in our investigation. First, the retrospective observational design inherently carries risks of selection bias and unmeasured confounding. Second, the absence of blinding during imaging interpretation introduces potential operator-dependent bias, particularly given the subjective components of PDL assessment. Future investigations would benefit from implementing independent core laboratory adjudication with pre-specified quantitative criteria to enhance reproducibility. Third, while demonstrating procedural feasibility, our single-center experience with limited sample size (*n* = 40) constrains external validity and statistical power—the cohort's insufficient magnitude precludes meaningful analysis of rare complications like device embolization (estimated prevalence <1%), despite the absence of such events in our series. Multicenter registries are recommended to establish definitive safety profiles through robust adverse event capture.

## Conclusions

The one-stop strategy provides a safe and efficient alternative for stroke prevention in AF patients with ASD, particularly those unsuitable for long-term anticoagulation. Larger studies are needed to validate long-term efficacy.

## Data Availability

The raw data supporting the conclusions of this article will be made available by the authors, without undue reservation.
